# Associations of healthy food choices with gut microbiota profiles

**DOI:** 10.1093/ajcn/nqab077

**Published:** 2021-05-21

**Authors:** Kari K Koponen, Aaro Salosensaari, Matti O Ruuskanen, Aki S Havulinna, Satu Männistö, Pekka Jousilahti, Joonatan Palmu, Rodolfo Salido, Karenina Sanders, Caitriona Brennan, Gregory C Humphrey, Jon G Sanders, Guillaume Meric, Susan Cheng, Michael Inouye, Mohit Jain, Teemu J Niiranen, Liisa M Valsta, Rob Knight, Veikko V Salomaa

**Affiliations:** Department of Food and Nutrition, University of Helsinki, Helsinki, Finland; Department of Public Health and Welfare, Finnish Institute for Health and Welfare, Helsinki, Finland; Department of Public Health and Welfare, Finnish Institute for Health and Welfare, Turku, Finland; Department of Future Technologies, University of Turku, Turku, Finland; Department of Public Health and Welfare, Finnish Institute for Health and Welfare, Helsinki, Finland; Department of Medicine, Turku University Hospital and University of Turku, Turku, Finland; Department of Public Health and Welfare, Finnish Institute for Health and Welfare, Helsinki, Finland; Institute for Molecular Medicine Finland, Helsinki, Finland; Department of Public Health and Welfare, Finnish Institute for Health and Welfare, Helsinki, Finland; Department of Public Health and Welfare, Finnish Institute for Health and Welfare, Helsinki, Finland; Department of Public Health and Welfare, Finnish Institute for Health and Welfare, Helsinki, Finland; Department of Public Health and Welfare, Finnish Institute for Health and Welfare, Turku, Finland; Department of Medicine, Turku University Hospital and University of Turku, Turku, Finland; Department of Pediatrics, University of California San Diego, La Jolla, CA, USA; Department of Pediatrics, University of California San Diego, La Jolla, CA, USA; Department of Pediatrics, University of California San Diego, La Jolla, CA, USA; Department of Pediatrics, University of California San Diego, La Jolla, CA, USA; Department of Pediatrics, University of California San Diego, La Jolla, CA, USA; Cornell Institute for Host-Microbe Interaction and Disease, Cornell University, Ithaca, NY, USA; Department of Infectious Diseases, Central Clinical School, Monash University, Melbourne, Australia; Cambridge Baker Systems Genomics Initiative, Baker Heart and Diabetes Institute, Melbourne, Australia; Division of Cardiology, Brigham and Women's Hospital, Boston, MA, USA; Smidt Heart Institute, Cedars-Sinai Medical Center, Los Angeles, CA, USA; Cedars-Sinai Medical Center, Los Angeles, CA, USA; Cambridge Baker Systems Genomics Initiative, Baker Heart and Diabetes Institute, Melbourne, Australia; Cambridge Baker Systems Genomics Initiative, Department of Public Health and Primary Care, University of Cambridge, Cambridge, United Kingdom; Departments of Medicine and Pharmacology, University of California San Diego, La Jolla, CA, USA; Department of Public Health and Welfare, Finnish Institute for Health and Welfare, Helsinki, Finland; Department of Public Health and Welfare, Finnish Institute for Health and Welfare, Turku, Finland; Department of Medicine, Turku University Hospital and University of Turku, Turku, Finland; Department of Public Health and Welfare, Finnish Institute for Health and Welfare, Helsinki, Finland; Department of Pediatrics, University of California San Diego, La Jolla, CA, USA; Department of Public Health and Welfare, Finnish Institute for Health and Welfare, Helsinki, Finland

**Keywords:** cross-sectional study, dietary score, epidemiology, healthy diet, metagenomics, microbiology, microbiota, nutrition

## Abstract

**Background:**

Diet has a major influence on the human gut microbiota, which has been linked to health and disease. However, epidemiological studies on associations of a healthy diet with the microbiota utilizing a whole-diet approach are still scant.

**Objectives:**

To assess associations between healthy food choices and human gut microbiota composition, and to determine the strength of association with functional potential.

**Methods:**

This population-based study sample consisted of 4930 participants (ages 25–74; 53% women) in the FINRISK 2002 study. Intakes of recommended foods were assessed using a food propensity questionnaire, and responses were transformed into healthy food choices (HFC) scores. Microbial diversity (alpha diversity) and compositional differences (beta diversity) and their associations with the HFC score and its components were assessed using linear regression. Multiple permutational multivariate ANOVAs were run from whole-metagenome shallow shotgun–sequenced samples. Associations between specific taxa and HFC were analyzed using linear regression. Functional associations were derived from Kyoto Encyclopedia of Genes and Genomes orthologies with linear regression models.

**Results:**

Both microbial alpha diversity (β/SD, 0.044; SE, 6.18 × 10^−5^; *P* = 2.21 × 10^−3^) and beta diversity (*R*^2^, 0.12; *P* ≤ 1.00 × 10^−3^) were associated with the HFC score. For alpha diversity, the strongest associations were observed for fiber-rich breads, poultry, fruits, and low-fat cheeses (all positive). For beta diversity, the most prominent associations were observed for vegetables, followed by berries and fruits. Genera with fiber-degrading and SCFA-producing capacities were positively associated with the HFC score. The HFC score was associated positively with functions such as SCFA metabolism and synthesis, and inversely with functions such as fatty acid biosynthesis and the sulfur relay system.

**Conclusions:**

Our results from a large, population-based survey confirm and extend findings of other, smaller-scale studies that plant- and fiber-rich dietary choices are associated with a more diverse and compositionally distinct microbiota, and with a greater potential to produce SCFAs.

See corresponding editorial on page 420.

## Introduction

What we eat is among the most influential environmental factors that determine long-term health (1, 2). Our everyday diet can increase or decrease the risk of noncommunicable conditions, such as cardiovascular diseases (3), metabolic syndrome (4), and cancer (2). Diet is also among the most important environmental factors that our microbial population in the gastrointestinal tract is exposed to and modified by daily (5, 6).

Gastrointestinal diseases (7), obesity (8), cardiovascular diseases (9), rheumatoid arthritis (10), and neurological disorders (11) have all been associated with the gut microbiota. Many of these disorders are also associated with diet. The gut microbiota has various functions beneficial to health, including immune maturation and homeostasis; vitamin biosynthesis; biotransformation of xenobiotics to more bioavailable, potentially antioxidative metabolites; and production of SCFAs (12, 13). SCFAs have been studied extensively, since they have been shown to offer many health benefits (14, 15).

Since different microbes have different optimal environments for growing and surviving, dietary choices can have a large influence on the composition and function of our gut microbiota. It has been shown that high intake of fiber and substitution of SFAs with PUFAs are protective factors (16–18). A diet rich in these factors has been widely recommended by health authorities (19–21). What remains to be determined is the role of the gut microbiota in these findings. Promising results have been observed for associations between the gut microbiota and food items such as whole-grain products (22), berries (23), nuts (24), and legumes (25). However, food items in the diet do not exist in a vacuum. Components that constitute a diet can have counteracting or synergistic effects with each other (26). Thus, the final assessment should always be made based on information that takes into account the entirety of the diet.

Research on microbiota-diet associations with a whole-diet focus is scarce. Previous studies in this domain have mainly focused on the Mediterranean diet (27) or various plant-based diets (28–31) in small and selected study samples. A larger study has assessed diet-microbiota associations in older, community-dwelling men by comparing prudent and Western diet patterns (32). No studies focusing on the whole diet with large, population-based samples have been conducted to date.

In the current study, we examined the associations between the gut microbiota and consumption of food items recommended to be part of a healthy diet in a cross-sectional setting, in a large, Finnish, population-based study sample. Our main objective was to assess whether healthy food choices, indicated by a summary score, are related to gut microbiota composition within samples (alpha diversity) and between samples (beta diversity). Furthermore, we assessed key bacterial taxa that have been previously identified as SCFA producers and their associations with healthy food choices. Finally, we performed a pathway analysis through Kyoto Encyclopedia of Genes and Genomes orthology (KO) groups to uncover the functional potential of the microbiota and how it associates with healthy food choices.

## Methods

We used the Strengthening the Reporting of Observational Studies in Epidemiology Statement (STROBE) cross-sectional reporting guidelines when writing this report (26).

### Study population

The National FINRISK Study originated from the North-Karelia project initiated in 1972 (33). It has been conducted by the Finnish Institute for Health and Welfare every 5 years until 2012 to assess risk factors for noncommunicable diseases, health behavior, and their changes in adult Finns.

The FINRISK 2002 cohort consists of 8738 individuals aged 25–74 years old who participated in the baseline examination. The exclusion criteria for this study were use of systemic antimicrobial medication within 4 months prior to the baseline examination (*n* = 1193); pregnancy at the time of baseline investigation (*n* = 47); incomplete records of nutritional, sociodemographic, or lifestyle information (*n* = 1549); or no available stool sample (*n* = 1019). The final study sample consisted of 4930 individuals (**Supplemental Figure 1**).

FINRISK 2002 was approved by the Ethical Committee on Epidemiology and Public Health of the Helsinki and Uusimaa Hospital District (decision number 87/2001), and the participants gave informed consent. The study was conducted according to the World Medical Association's Declaration of Helsinki on ethical principles (34).

### Covariates

FINRISK 2002 included a questionnaire and a health examination. Questions on sociodemographic and lifestyle factors were answered prior to the clinical examination by filling out a questionnaire enclosed with the invitation letter. These questionnaires were brought to the health examination and inspected by trained nurses.

Based on prior literature, we selected 5 covariates: age, sex, BMI, smoking status, and usage of microbiota-altering medications. Stages of covariate selection, from demographic only to fuller models, have been listed in **Supplemental Table 1**. BMI was calculated as weight (kg) divided by height (m) squared. Weight and height were measured at the clinic according to standard international protocols with light clothing and without shoes (35). The participants were divided into 2 groups by smoking status: current smokers and nonsmokers who had not smoked in the last 6 months. In addition to the excluded systemic antimicrobial medicines, a variety of other drugs can potentially affect the microbiota. To account for this, we created a dummy variable where participants were divided into users and nonusers. A listing of the drugs and the systemic antimicrobial medications is presented in **Supplemental Methods**. Information on the usage of these drugs was acquired from a register on prescribed medicine purchases maintained by the Social Insurance Institution of Finland (36). A participant was flagged as a user if they had a registered purchase within 3 months prior to the study. Over-the-counter medicines are not included in the register and are therefore missing from the data. The records were linked with the study data using unique national personal identity numbers given to each permanent resident in Finland.

### Dietary information

Habitual diet was assessed using a food propensity questionnaire (FPQ) that contained 42 food items with 6 choices of consumption frequency. The choices were interpreted with the assumptions that a month consists of 30 days, a week consists of 7 days, and a month consists of 4.3 weeks (30/7). Answers were converted to times-per-month values as follows: an answer of 1 (less than once a month) was converted to 0.5 times per month; 2 (once or twice a month) to 1.5 times per month; 3 (once a week) to 4.3 times per month; 4 (couple of times a week) to 8.6 times per month (interpreted as twice a week); 5 (almost every day) to 21.5 times per month (interpreted as 5 times a week); and finally 6 (once a day or more often) to 30, 45, or 60 times per month using the following principle. Food items that are rarely eaten more than once a day, such as sausages, meat dishes, and so forth, were given the value of 30 times per month. Food items that are often eaten multiple times a day, such as fresh vegetables, breads, and so forth, were given a value of 60 times per month. Food items that fall in between these 2 groups were given 45 points. The scoring of responses to consumption of red and processed meat products was done in an inverse manner (i.e., a response of “almost every day” would convert to 0.5, etc.) to account for their less favorable role compared to the other components in the score. Scoring for food items used in the final analyses is shown in **Table 1**.

**TABLE 1:** Summary of dietary components, their food items, and score ranges

Components	Score range^1^	Constituting food items
Breads	1–120	Rye and crisp bread^2^
		Graham and multi-grain bread^2^
Vegetables	1.5–150	Fresh vegetables and root vegetables^2^
		Cooked vegetables and legumes^3^
		Vegetable dishes^3^
Fruits	0.5–60	Fruits^2^
Berries	0.5–45	Fresh and frozen berries^3^
Juices	0.5–45	Fruit and berry juices^3^
Fish	0.5–45	Fish, fish products, and fish dishes^3^
Poultry	0.5–45	Poultry, poultry products, and poultry dishes^3^
Low-fat cheeses	0.5–60	Low-fat cheeses^2^
Dressings and oils	0.5–45	Salad dressings and oils^3^
Nuts and seeds	1–90	Nuts^3^
		Seeds^3^
Red and processed meat products	2–150	Meat dishes^4^
		Sausages^4^
		Cold cuts^3^
		Charcuterie^3^

1 Each component's possible range is displayed as times per month.

^2^The scores for individual food items range from 0.5 to 60 points.

^3^The scores for individual food items range from 0.5 to 45 points.

^4^The scores for individual food items range from 0.5 to 30 points.

A healthy food choices (HFC) score was formed by choosing and summing FPQ responses to food items that are recommended in the Nordic Nutrition Recommendations dietary guidelines as part of a healthy diet (19). Food items chosen to be components of the score were fiber-rich breads; vegetables (including beans and lentils); fruits; berries; fresh, nonsweetened berry and fruit juices; fish; poultry; low-fat cheeses; salad dressings and oils; and nuts and seeds. The HFC score effectively acts as an indicator for an omnivorous Nordic diet rich in plants, fiber, and PUFAs.

Based on the consumption of the constituent components, the HFC score ranges from 9–745, where a higher score represents a greater number of healthy food choices per month. The score was calculated by summing the transformed monthly consumption scores for all chosen components. A summary of the HFC score's structure and a listing of each components’ respective constituent food items are displayed in Table 1. Additionally, a combined fiber sources score was created to combine all food components that are sources of dietary fiber into 1 summary variable. This score was a simple sum of the consumption frequencies of all related food items: fiber-rich breads, vegetables, fruits, berries, and fresh fruit and berry juices (these include products such as berry nectars, fruit juices that include the pulp, etc.).

### Stool samples

All participants were asked to donate a stool sample. Those willing to do so were given instructions and equipment to gather a stool sample at home, then send it overnight between Monday and Thursday to the study personnel using prepaid postal parcels under typical Finnish winter conditions. The samples were collected into 50 ml Falcon tubes without a stabilizing solution. Sample tubes were preidentified with the participants’ respective study IDs and frozen immediately upon reception. The samples were stored unthawed at −20°C until sequencing.

Metagenomic data were based on whole-genome, untargeted shallow shotgun sequencing, analyzed at the University of California, San Diego, California (37). The samples were normalized to 5-ng inputs using an Echo 550 acoustic liquid handling robot and were sequenced using Illumina Hi-Seq 4000 (Illumina Inc., San Diego, CA, USA) for paired-end 150-bp reads. The average read count was approximately 900,000 reads per sample. A more detailed description of protocols for DNA extraction and library preparation has been reported elsewhere (38). Classification and assigning of raw sequence data into microbial taxa were performed using SHallow shOtGUN profiler (SHOGUN) v1.0.5 (Knights Lab, University of Minnesota, Minneapolis, MN, USA) (39) against a database of complete archaeal, bacterial, and viral genomes in NCBI Reference Sequence Database (NCBI RefSeq) v82 [National Center for Biotechnology Information (NCBI), U.S. National Library of Medicine, Bethesda, MD, USA; May 8, 2017]. The classified microbial data were used in the compositional data form. Batch variables were not accounted for in the analyses.

In per taxa analyses, the taxa were filtered down to a core microbiota that included any genus with a minimum abundance of 0.1% and a prevalence of at least 1% across all samples, similar to the filtering thresholds used by Salosensaari et al. (38). Bacterial species with the potential to produce SCFAs were identified based on a review of the current literature (40–42). Species that were classified and present in the samples were selected for per-taxa analyses on the species level. These species were *Akkermansia muciniphila, Faecalibacterium prausnitzii*, and *Roseburia intestinalis*.

### Statistical methods

Alpha diversity is a measure that quantifies intra-individual diversity of the microbiota and acts as a rough indicator of the overall species richness of a single individual. Beta diversity quantifies inter-individual diversity and gives information on the differences of microbiotas between individuals, thus acting as a measure of composition. In this study, we quantified alpha diversity using the Shannon index and beta diversity using Bray-Curtis dissimilarity scores (43, 44). All analyses were adjusted for age, sex, BMI, smoking, and use of potentially microbiota-altering medication. Interaction effects of the HFC score with sex and age were not statistically significant, and thus were excluded from the final analyses.

We assessed the associations between alpha diversity and diet using linear regression. Principal coordinate analysis (PCA), permutational multivariate analysis of variance (PERMANOVA), distance-based redundancy analysis (dbRDA) and analysis of similarities (ANOSIM) were used to analyze beta diversity (45–47). PCA, in conjunction with ANOSIM, was used to assess clustering of samples; PERMANOVA was used to assess the amount of variance each variable can explain in the distances between the samples; and finally dbRDA was used to discover the direction that each of those variables take for that variance. A dbRDA is distinct from a PCA in that it is a constrained ordination method that displays and explains variation in a set of response variables that are constrained by a set of predictor variables, effectively linking multivariate regression analysis and PCA (46). The constrained variance in a dbRDA is the portion of total variance in the set of response variables (the Bray-Curtis distances of the samples in this case) that can be explained by the provided set of predictor variables.

PERMANOVA, dbRDA, and ANOSIM were all run with 999 permutations. For per taxa analyses, an analysis tool called MaAsLin (multivariate association with linear models) was used (48). We used the tool to run a series of multivariate linear regression models with adjustments made for covariates and multiple comparisons. The relative abundances of the taxa were arcsine square root–transformed prior to analysis. In the models, the abundances were used as dependent variables and dietary elements as independent variables. Each MaAsLin run produced results for associations between all taxa and the chosen dietary element. A pathway analysis was done by associating KO groups with the HFC score in linear regression models. The relative abundances of KO groups for each sample were gathered from the strain-level outputs of SHOGUN. KO group data were standardized with a log10 transformation prior to analysis, and only statistically significant associations were selected and visualized using FuncTree (Yamada Lab, Tokyo Institute of Technology, Tokyo, Japan) (49). Separate plots were made for estimates that had positive and negative associations.

Alpha diversity, beta diversity, and per taxa analyses were all done for the HFC score as a whole, as well as for its individual components. A pathway analysis was done only for the HFC score. The estimates given by regression models for alpha diversity and for the per taxa analyses were standardized per 1 SD. The level of statistical significance for all analyses except the per taxa analyses was set at a 2-sided *P* value < 0.05. For per taxa analyses, a Benjamini-Hochberg false discovery rate–corrected *Q*-value < 0.05 was used for the *P* values obtained from the linear models assessing the taxa-diet associations.

The primary outcome variables for this study were associations of the HFC score with *1*) the Shannon alpha diversity measure; *2*) Bray-Curtis dissimilarity scores; *3*) specific taxa abundances; and *4*) KO groups. Secondary outcome variables were associations of the dietary components of the HFC score with the same listed variables, excluding KO groups.

All statistical analyses were performed using R version 3.6.1 (R Core Team, Vienna, Austria) (50). The *phyloseq, microbiome*, and *vegan* packages were central to the statistical analyses (51–53).

## Results

### Descriptive statistics

Detailed characteristics for the study sample are displayed in **Table 2**. The average age of the participants was 48 years, with a slight overrepresentation of women (53%). The average BMI of the participants was 26.9 kg/m^2^; 37.1% used potentially microbiota-altering medication; and 23.7% were current smokers. Women tended to have a higher HFC score compared to men (217.8 ± 90.6/mo compared with 176.9 ± 80.4/mo, respectively). Sex differences were especially notable for intakes of vegetables (15.2/mo higher for women), fruits (10.1/mo higher for women), red and processed meat products (8.6/mo higher for men), low-fat cheeses (7.4/mo higher for women), and berries (3.9/mo higher for women).

**TABLE 2:** Descriptive characteristics of the study sample

	Men (*n* = 2311)		Women (*n* = 2619)		All (*n* = 4930)	
Variable	Mean ± SD	Median (IQR)	Mean ± SD	Median (IQR)	Mean ± SD	Median (IQR)
Age, y	49 ± 12.8	—	47 ± 12.8	—	48 ± 12.8	—
BMI, kg/m^2^	27.3 ± 4.1	—	26.5 ± 5.0	—	26.9 ± 4.6	—
Medication users, *n*	680 (29.4%)	—	1151 (43.9%)	—	1831 (37.1%)	—
Current smokers, *n*	659 (28.5%)	—	510 (19.5%)	—	1169 (23.7%)	—
HFC score, 1/mo	219.2 ± 91.1	203.6 (116.9)	277.2 ± 105.6	266.3 (151.4)	250.0 ± 103.2	234.9 (144.1)
Breads, 1/mo	64.1 ± 29.6	64.3 (38.5)	65.5 ± 30.4	64.3 (38.5)	64.8 ± 30.0	64.3 (38.5)
Vegetables, 1/mo	33.3 ± 29.8	24.5 (36.7)	48.5 ± 35.2	38.7 (48.6)	41.4 ± 33.7	31.1 (50.1)
Fruits, 1/mo	20.2 ± 20.1	8.6 (17.2)	30.3 ± 23.0	21.5 (51.4)	25.6 ± 22.3	21.5 (51.4)
Berries, 1/mo	7.9 ± 11.0	4.3 (7.1)	11.8 ± 13.4	8.6 (20.0)	10.0 ± 12.5	4.3 (7.1)
Fruit and berry juices, 1/mo	15.7 ± 15.5	8.6 (20.0)	16.4 ± 15.8	8.6 (20.0)	16.1 ± 15.7	8.6 (20.0)
Fish, 1/mo	5.7 ± 5.6	4.3 (7.1)	5.5 ± 4.8	4.3 (7.1)	5.6 ± 5.2	4.3 (7.1)
Red and processed meat products, 1/mo	40.0 ± 23.3	35.9 (32.1)	31.4 ± 22.0	27.8 (28.6)	35.4 ± 23.0	32.1 (31.6)
Poultry, 1/mo	4.6 ± 4.5	4.3 (7.1)	5.4 ± 4.8	4.3 (7.1)	5.0 ± 4.7,	4.3 (7.1)
Low-fat cheeses, 1/mo	13.6 ± 19.2	4.3 (21.0)	21.0 ± 23.0	8.6 (20.0)	17.6 ± 21.6	8.6 (20.0)
Dressings and oils, 1/mo	6.6 ± 10.0	1.5 (8.1)	7.5 ± 10.8	1.5 (8.1)	7.1 ± 10.5	1.5 (8.1)
Nuts and seeds, 1/mo	2.4 ± 6.0	1.0 (0)	3.3 ± 7.7	1.0 (1.0)	2.8 ± 7.0	1.0 (1.0)

Medication users are individuals who used potentially microbiota-altering medication (listed in Supplemental Methods) within 3 months prior to the examination. Current smokers are individuals who smoked within 6 months prior to the examination. Values are means ± 1 SD (excluding medication users and current smokers), followed by the median, with the IQR in parenthesis for nutritional variables. Units for the dietary components are the respective consumption frequencies for each item as times per month. The HFC score was calculated by first transforming original FPQ responses to times-per-month values and then summing these values for food items that are regarded as being part of a healthy diet. The times-per-month values for red and processed meat products were inverted prior to adding them to the HFC score, to account for their negative roles in the diet. Abbreviations: FPQ, food propensity questionnaire; HFC, healthy food choices.

To check the representativeness of our population sample, we compared the characteristics of those individuals who did not donate a stool sample (*n* = 1019) to those included in this study (*n* = 4930). The groups differed significantly in all compared variables: age, sex, BMI, smoking, medication usage, and the HFC score. The group that did not donate a sample was younger, was comprised of more men, had slightly lower BMI on average, was comprised of more smokers and fewer medication users, and the individuals had a lower HFC score on average (**Supplemental Table 2**).

### Microbial diversity

**FIGURE 1:**
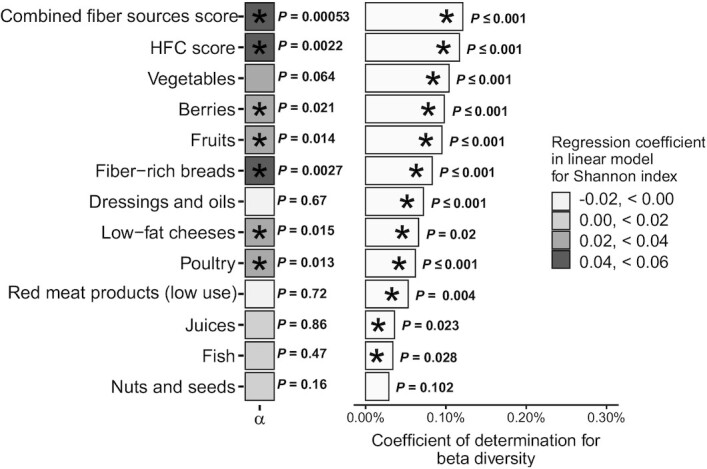
Alpha and beta diversity results (*n* = 4930). The HFC score is a summary variable that consists of the summed monthly consumption frequencies of the individual food components listed beneath it. Red and processed meat products have inverse grading compared to those of other components to account for their negative impact on diet quality; thus, a higher score implies low use of such products. Combined fiber sources score is a summary variable that includes only food components that are sources of dietary fiber. Alpha diversity (Shannon index; mean, 3.44; SD, 0.41) on the left was analyzed using linear regression models and was standardized per SD. The shading of the boxes on the left correspond to the strength of the association. PERMANOVA results (R^2^) for beta diversity (Bray-Curtis dissimilarity) are on the right. Both analyses were adjusted for age, sex, BMI, and use of potentially microbiota-altering medication within 3 months prior to study. *Statistically significant results (*P* value < 0.05), with the *P* value labeled on the right. Abbreviations: HFC, healthy food choices; PERMANOVA, permutational multivariate analysis of variance.

The study sample had an average Shannon alpha diversity measure of 3.44 and an SD of 0.41. The measure was statistically significant in a multiple linear regression model with the Shannon alpha diversity index as the dependent variable and the HFC score as the independent variable. Baseline data on age, sex, BMI, smoking, and use of microbiota-altering medications were used as covariates. Alpha diversity increased approximately 0.044 points per 1 SD change in the HFC score (*P* = 2.21 × 10^−3^; **Figure 1**; **Table 3**). Covariate effects per 1 SD (β/SD) for predicted alpha diversity were 0.054 (age; *P* = 1.42 × 10^−4^), −0.039 (sex; coded as females = 0, males = 1; *P* = 6.29 × 10^−3^), −0.082 (BMI; *P* = 7.60 × 10^−9^), −0.049 (smoking; *P* = 6.03 × 10^−4^), and −0.039 (medication use; *P* = 5.71 × 10^−3^). For a more comprehensive listing of results, along with CIs, see **Supplemental Table 3**.

**TABLE 3:** Results of linear regression models predicting Shannon alpha diversity measure

Variable	β/SD	SE	*P* value
HFC score	0.044	6.18 × 10^−5^	2.21 × 10^−3^
Combined fiber sources score	0.049	8.75 × 10^−5^	5.31 × 10^−4^
Fiber-rich breads	0.043	1.97 × 10^−4^	2.70 × 10^−3^
Poultry	0.036	1.26 × 10^−3^	1.28 × 10^−2^
Fruits	0.035	2.73 × 10^−4^	1.44 × 10^−2^
Low-fat cheeses	0.035	2.75 × 10^−4^	1.51 × 10^−2^
Berries	0.033	4.98 × 10^−4^	2.12 × 10^−2^
Vegetables	0.026	1.80 × 10^−4^	6.38 × 10^−2^
Fruit and berry juices	0.0025	3.72 × 10^−4^	8.59 × 10^−1^
Nuts and seeds	0.020	8.39 × 10^−4^	1.64 × 10^−1^
Fish	0.010	1.15 × 10^−3^	4.65 × 10^−1^
Red and processed meat products (low use)	−0.0051	1.50 × 10^−4^	7.21 × 10^−1^
Dressings and oils	−0.0062	5.57 × 10^−4^	6.66 × 10^−1^

Each row represents the results of one regression model, sorted by standardized effect strength with the HFC score on the first row and combined fiber sources score on the second row. The scores’ individual components are listed starting from row three. Covariates in each model include age, sex, BMI, smoking, and potentially microbiota-altering medication. Interaction effects were nonsignificant and thus were omitted from the analyses. Abbreviations: HFC, healthy food choices.

The score for combined fiber sources had a positive association with alpha diversity (β/SD, 0.049; *P* = 5.31 × 10^−4^). The strongest positive associations between alpha diversity and HFC score components were observed for fiber-rich breads, followed by poultry, fruits, low-fat cheeses, and berries (Table 3). No statistically significant negative associations were observed for any score components.

### Microbial composition

Beta diversity had statistically significant associations with the HFC score (PERMANOVA *R*^2^ = 0.12; *P* ≤ 1.00 × 10^−3^; Figure 1) when controlling for age, sex, BMI, smoking, and medication use. Another analysis where all the components of the HFC score were included together returned an *R*^2^ of 0.60 (*P* ≤ 1.00 × 10^−3^). HFC score components had the following statistically significant associations with microbiota composition in analyses where they were the sole dietary variable: vegetables (*R*^2^ = 0.10; *P* ≤ 1.00 × 10^−3^), berries (*R*^2^ = 0.098; *P* ≤ 1.00 × 10^−3^), fruits (*R*^2^ = 0.095; *P* ≤ 1.00 × 10^−3^), fiber-rich breads (*R*^2^ = 0.083; *P* ≤ 1.00 × 10^−3^), dressings and oils (*R*^2^ = 0.072; *P* ≤ 1.00 × 10^−3^), low-fat cheeses (*R*^2^ = 0.066; *P* = 2.00 × 10^−3^), poultry (*R*^2^ = 0.062; *P* ≤ 1.00 × 10^−3^), lower use of red and processed meat products (*R*^2^ = 0.0053; *P* = 4.00 × 10^−3^), fresh fruit and berry juices (*R*^2^ = 0.036; *P* = 2.30 × 10^−2^), and fish (*R*^2^ = 0.034; *P* = 2.80 × 10^−2^). Nuts and seeds had no statistically significant associations. The score for combined fiber sources had a slightly stronger association (*R*^2^ = 0.12; *P* ≤ 1.00 × 10^−3^) with beta diversity than the HFC score.

**FIGURE 2:**
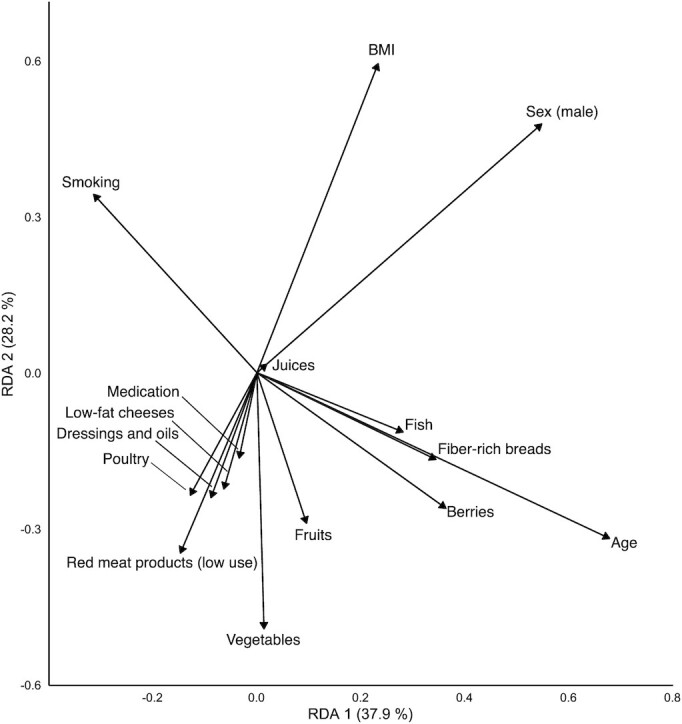
Distance-based redundancy analysis results for the HFC score components, explaining the variance in beta diversity results (*n* = 4930). The analysis explains variation in the distances between Bray-Curtis dissimilarity scores of the samples by constraining their ordination with a set of explanatory variables (unlike in a principal coordinate analysis, where the ordination is unconstrained). Directions of the vectors display directions of associations for the covariates and components of the HFC score with Bray-Curtis dissimilarity scores on the first 2 axes of the ordination. The lengths of the vectors correspond to the strength of correlation. The further away an item is from the origin, the greater its contribution to variance. The closer 2 items are to each other, the more similar their effect on variance. The amount of constrained variance (i.e., the percentage of variance explainable by the current set of explanatory variables) explained by axes RDA1 and RDA2 is displayed in parenthesis on the respective axis next to the axis label. Abbreviations: HFC, healthy food choices; RDA, redundancy analysis.

A dbRDA was performed to determine the directions of these associations. The analysis included all individual components of the HFC score that had a statistically significant association with beta diversity, as well as all the previously mentioned covariates. The result was significant (*P* ≤ 1.00 × 10^−3^), with 1.47% of total variance explained (**Supplemental Table 4**). The first 2 axes accounted for 66% of the constrained variance and 0.92% of the total variance. A biplot of the ordination on the first 2 axes is displayed in **Figure 2**. Qualitative interpretation of the plot revealed that the vector directions on the second axis divide the variables into 2 distinct groups. All components of the HFC score, except for fresh, nonsweetened berry and fruit juices, along with use of potentially microbiota-altering medication and age, pointed downwards and were associated with the second axis in an opposite manner to the components of BMI, male sex, and smoking.

**FIGURE 3:**
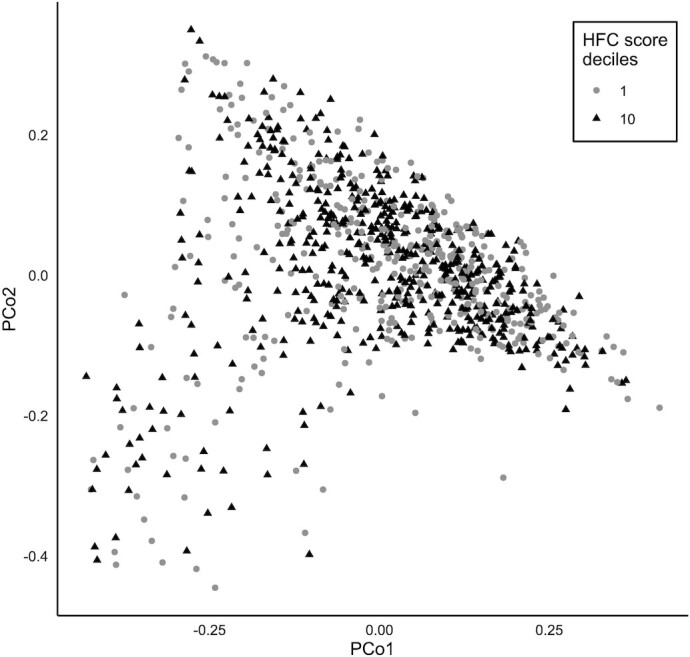
Principal coordinate analysis plot depicting the ordination of Bray-Curtis dissimilarity scores of the individuals in the first and last deciles (*n* = 986) of the HFC score on the first 2 principal coordinates. The closer 2 points are, the more similar their microbiotas are. Abbreviations: HFC, healthy food choices; PCo, principal coordinate analysis.

A PCA was performed using the Bray-Curtis dissimilarity score. The first 2 principal coordinates are displayed in **Figure 3** with samples of the 2 extreme deciles of the HFC score. No clear clustering of microbiotas that would be explained by the HFC score was detectable by visual inspection of the plot. However, a statistically significant ANOSIM test (*R* = 0.0288; *P* ≤ 1.00 × 10^−3^) revealed that individuals in the 2 extreme deciles of the HFC score harbored compositionally distinct microbiotas.

### Per taxa analysis

**FIGURE 4:**
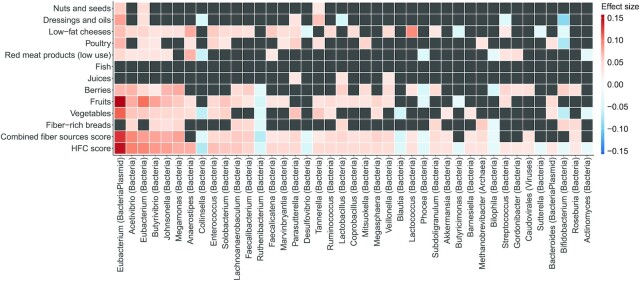
MaAsLin analysis results for the HFC score, combined fiber sources score, and their constituting elements, filtered by core genera and sorted by effect size (*n* = 4930). Microbial abundances were arcsine square root–transformed, and the analyses were adjusted for age, sex, BMI, and use of potentially microbiota-altering medication within 3 months prior to study. Each square represents the effect strength per SD (β/SD) of a linear regression model, run between the respective dietary component or score and bacterial genus. Significance threshold was a false discovery rate–corrected *Q*-value < 0.05. A dark gray square indicates a nonsignificant result. Abbreviations: HFC, healthy food choices; MaAsLin, multivariate association with linear models.

The stool samples contained a total of 5748 species in 2019 genera before filtering to the core microbiota. A visualization of prevalences of the core microbiota at the genus level is shown in **Supplemental Figure 2**. The core microbiota consisted of bacteria, bacterial plasmids, archaea, and viruses in 91 genera, of which 75 had at least 1 statistically significant association with a dietary component or score. Of these, 41 genera had statistically significant associations with the HFC score (**Figure 4**; for a comprehensive view, see **Supplemental Figure 3** and **Supplemental Table 5**).

The top 10 assigned taxa on the genus level that were associated with the HFC score were *Eubacterium* plasmids (β/SD, 0.15; *q* = 4.37 × 10^−23^), *Acetivibrio* (β/SD, 0.097; *q* = 8.15 × 10^−11^), *Eubacterium* (β/SD, 0.095; *q* = 2.43 × 10^−10^), *Butyrivibrio* (β/SD, 0.085; *q* = 1.28 × 10^−8^), *Johnsonella* (β/SD, 0.082; *q* = 4.65 × 10^−8^), *Megamonas* (β/SD, 0.075; *q* = 2.68 × 10^−6^), *Anaerostipes* (β/SD, 0.074; *q* = 8.27 × 10^−7^), *Collinsella* (β/SD, −0.071; *q* = 2.18 × 10^−6^), *Enterococcus* (β/SD, 0.065; *q* = 1.31 × 10^−5^), and *Solobacterium* (β/SD, 0.061; *q* = 5.35 × 10^−5^). All components of the HFC score had statistically significant independent associations with at least 1 core genus (Supplemental Table 5).

In a species-level analyses, all of the selected species (see “Stool samples” above) had statistically significant associations with the HFC score: *Faecalibacterium prausnitzii* (β/SD, 0.050; *q* = 5.34 × 10^−4^), *Akkermansia muciniphila* (β/SD, 0.037; *q* = 1.17 × 10^−2^), and *Roseburia intestinalis* (β/SD, 0.032; *q* = 2.29 × 10^−2^).

### Pathway analysis

The samples contained 5968 KO groups in total. Of these, 788 KO groups had a statistically significant association with the HFC score. Positive associations were observed with 657 KO groups and negative associations were observed with 131 groups (**Supplemental Table 6**).

**FIGURE 5:**
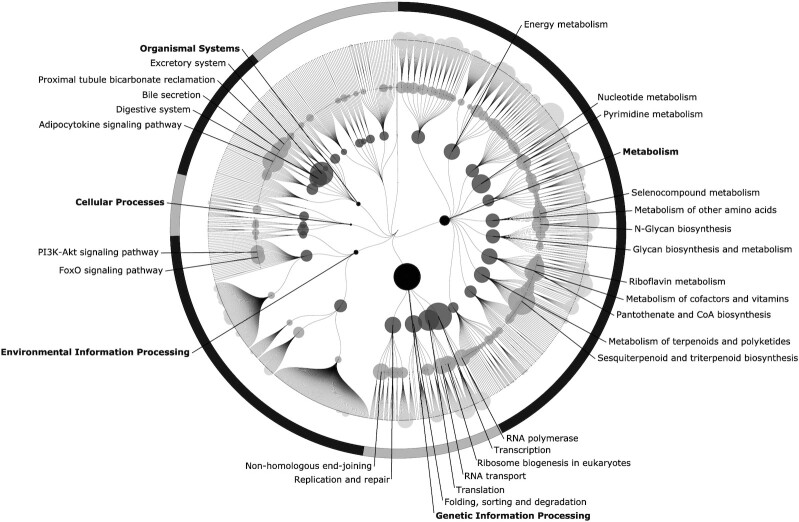
Positive associations of functional pathways in the gut microbiota with the HFC score (*n* = 4930). The layers from darker to lighter are biological categories (very dark gray), biological processes (dark gray), pathways (medium gray), and modules (light gray). Node size is determined by the average value of all the estimates of statistically significant (*P* value < 0.05) KO groups assignable to that node. Estimates were calculated with linear regression models, with each KO group being the dependent variable. The HFC score, along with age, sex, BMI, smoking, and use of potentially microbiota-altering medication within 3 months prior to the study, were used as independent variables. For clarity, node labels for only the 3 highest layers were included. Displayed labels were filtered to only include nodes which had a size greater than 150. Abbreviations: FoxO, Forkhead box protein class O; HFC, healthy food choices, KO, Kyoto Encyclopedia of Genes and Genomes orthology; PI3K-Akt, Phosphoinositide 3-kinase - Protein kinase B signalling pathway.

Most prominent positive associations were observed in the functional category of genetic information processing, in processes such as transcription, translation, protein folding, sorting and degradation, and DNA replication and repair (**Figure 5**). Enrichment of the SCFA metabolism was observed as well. Statistically significant associations were observed for KO groups involved in both butyrate and propionate metabolisms (Supplemental Table 6). Other notable statistically significant associations were observed with KO groups involved in the biosynthesis of N-glycan and vitamins, such as pantothenate and riboflavin, as well as adipocytokine signaling and bicarbonate reclamation functions.

**FIGURE 6:**
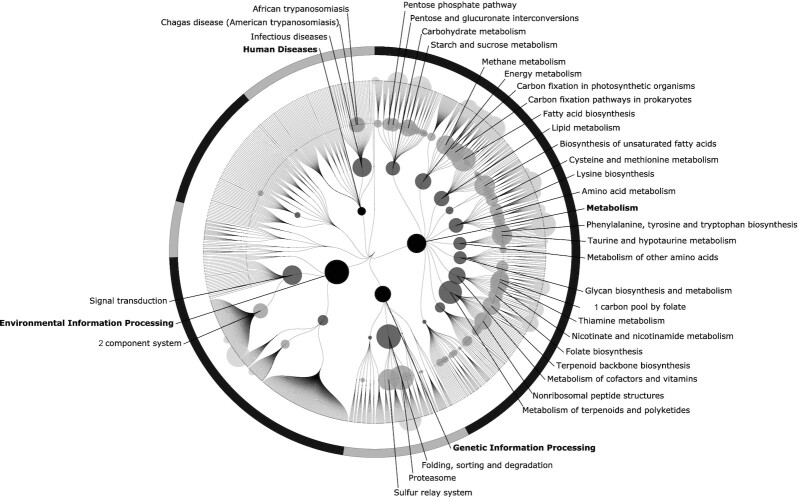
Inverse associations of functional pathways in the gut microbiota with the HFC score (*n* = 4930). Abbreviations: HFC, healthy food choices.

The most prominent negative associations were observed in pathways for proteasomes, fatty acid biosynthesis, the sulfur relay system, taurine, and the hypotaurine metabolism (**Figure 6**). Various pathways involved in the metabolism of vitamins, such as thiamine, folate, nicotinate, and nicotinamide, were also observed to be negatively associated with the HFC score. It is also noteworthy that there was an inverse association with the KO group cluster of biological processes in infectious diseases; upon closer inspection, this was due to the KO group for the enzyme oligopeptidase B, specific to *Escherichia coli* (54).

## Discussion

Our study used a whole-diet approach to assess associations between the human gut microbiota and healthy food choices in a large, population-based study. It offers new perspectives to findings of smaller studies, presents new insights into how healthy food items are associated with composition of the microbiota, and presents novel looks into the functional potential of the microbiota.

The HFC summary score we used was a significant predictor for the diversity and composition of the gut microbiota, although the associations were fairly weak. Healthy food choices were associated with a richer and compositionally distinct microbiota. Other comparable studies made in Western countries have found similar but also divergent results. A recent study found that high adherence to a healthy Mediterranean diet rich in plants was associated with higher levels of fecal SCFAs and lower levels of urinary trimethylamine N-oxide in 153 Italians (27). Similar to our findings, that study did not observe any clear clustering of the microbiotas based on diet, but instead discovered a gradual shift in composition. However, no statistically significant associations of alpha or beta diversity measures with diet were detected. Conversely, another cross-sectional study of 101 Italian individuals comparing omnivores with individuals eating a vegetarian or vegan diet found that vegetarians harbored microbiotas that were richer in alpha diversity compared to omnivores (31). Microbiota composition between the 3 groups was similar, which was hypothesized to be due to similar nutrient compositions of the diets. A third study, looking at diet-microbiota associations by comparing a Western and a prudent diet pattern in 517 elderly, community-dwelling men, found no connection between alpha diversity and the diet, but did note a significant association with beta diversity (32). As was demonstrated in our study, the associations between diversity and diet are very modest, and discrepancies in past results might be due to small sample sizes and/or study samples that were not representative of the whole population.

Our and others’ results indicate that dietary fiber is among the most significant dietary influencers of the gut microbiota (55). Associations have been observed with genera that include species with fiber-degrading and/or SCFA-producing capabilities such as *Eubacterium, Butyrivibrio, Ruminococcus, Faecalibacterium*, and *Roseburia* (40–42, 56). *Faecalibacterium* was positively associated with a prudent diet pattern in the aforementioned study in elderly men, while *Eubacterium* and *Ruminococcus* were associated with a Western diet pattern (32). It is important to note, however, that this particular study was conducted in the United States, while ours included European participants, thus making direct comparisons more difficult due to geographical differences in the microbial compositions of the communities (57). Furthermore, the levels of most commonly known SCFA-producing species, *Faecalibacterium prausnitzii, Akkermansia muciniphila*, and *Roseburia intestinalis*, were all significantly elevated in individuals with a higher HFC score in our study (40–42). These associations were accompanied by enrichment of enzymes involved in the SCFA metabolism, as well. These findings indicate that healthy dietary choices are indeed associated with a human gut microbiota that possesses a greater potential for SCFA synthesis. Species of the *Eubacterium,Ruminococcus*, and *Roseburia* genera, along with levels of SCFAs, have been previously identified to be more abundant in individuals consuming a plant-based diet (58). The study compared microbiota changes in a crossover setting in young American volunteers who consumed a plant-based and an animal-based diet ad libitum for 5 days each. *Bacteroides*, a bile-tolerant genus associated with an increased colorectal cancer risk (59), was conversely enriched in individuals consuming the animal-based diet. Notably, in our study, *Bacteroides* had a negative association with the summary score for combined fiber sources, which nevertheless disappeared once we looked at associations on the whole-diet level. The inverse association of the combined fiber sources score with *Bacteroides* supports other, similar findings between fiber intake and a reduced colorectal cancer risk (60).

The associations between red and processed meat products and the gut microbiome cannot be ignored either. The fact that diminished use of red and processed meat products correlated strongly in the same direction with other healthy components in our dbRDA indicates that increased usage of red and processed meat is associated with the microbiota composition in an opposite manner to that of a healthy diet. This is not surprising given that low levels of fiber and increased usage of red meat products have been linked repeatedly with dysbiosis of the microbiota and colorectal cancer (61). The HFC score is also associated negatively with enzymes involved in the metabolism of taurine, a major constituent of bile. This hints at diminished exposure to bile acids of the gut microbiota in individuals who have a healthier diet, which is possibly due to diminished use of red and processed meat products. Secondary bile acids produced by the microbiota are known contributors to the colorectal cancer risk (62). Notably, in our study, enzymes for the amino acid metabolism and the sulfur relay system were also negatively associated with the HFC score.

A major strength of our study is the large number of participants, constituting a large, population-based sample. In addition, the participants in our study were carefully phenotyped and apparently healthy. Another strength is the use of whole metagenomic shallow sequencing, which offers much more robust information taxonomically and functionally when compared with 16S RNA amplicon sequencing (37). However, the generalizability of our results is likely jeopardized by geographical differences in gut microbiota composition. The taxa-diet responses might be different in different sociocultural, economical, ethnic, and environmental settings (57). Additionally, the statistically significant differences between those who chose not to donate a stool sample and those included in the study suggest that our participants represent the more health-conscious part of the population, with healthier diets and lifestyles than nonparticipants. We want to point out, however, that our participation rate was high and any bias due to the healthy participant effect is likely to be small. Nevertheless, there exists a need to describe these links in different cohorts around the globe. Also, physical activity and alcohol consumption both have been noted to influence the gut microbiota, but were not taken into account in this study (63, 64).

Another limitation of our study, as in all studies with self-reported data, is the accuracy of the FPQ responses. Furthermore, the cross-sectional design of the study limits our ability to move beyond inferring associations. Additionally, we used a new diet score that does not encompass all the healthy diet guidelines presented in the Nordic Nutrition Recommendations, such as recommendations for salt intake (19). Medication data was also limited to only prescription drugs. However, as has been demonstrated, our results are in line with previous findings, which indicate that the HFC score can effectively quantify dietary habits. Furthermore, a simple healthy diet score representing a similar diet was found to be protective from coronary artery disease in genetically predisposed individuals in 3 prospective cohorts involving 55,685 individuals (65).

Taxonomic classification using shallow-sequenced metagenomes is a source of uncertainty. Correct classification of the microbes relies on the accuracy of the used database, which still gives varying results at the species level depending on the database used. Also, while shallow shotgun sequencing may be superior in capturing taxonomic diversity and functional characteristics when compared to 16S RNA amplicon sequencing, it is not as accurate as deep sequencing for capturing genetic features, which is why interpretation of the functional results should be done with some caution (66).

In conclusion, we determined that a recommended diet rich in plants, fiber, and PUFAs is associated with a more diverse and compositionally distinct individual microbiota in the gut. We further determined multiple taxa of interest that have associations with specific components of the diet. Especially noteworthy are the positive associations of a healthy diet with fiber-degrading and SCFA-producing species, which were accompanied by enrichment of enzymes involved in the SCFA metabolism. Thus, a healthy diet is associated with a greater potential for a SCFA-producing gut environment. The results of our study support the balanced plant-rich diet recommended by dieticians around the world, and warrant further study into more detailed effects of the diet on the human gut microbiota, especially on the species level, and its synergy with health and disease.

## Supplementary Material

nqab077_Supplemental_Files

## Data Availability

Data described in the manuscript, code book, and analytic code are available from Finnish Institute for Health and Welfare Biobank based on a written application as instructed on the website of the Biobank (https://thl.fi/en/web/thl‐biobank). The data are not publicly available because they contain information that could compromise research participant privacy/consent. The source code for the analyses is openly available at 10.5281/zenodo.4671405.

## Notes

The authors reported no funding received for this study.

The study was designed, implemented, analyzed, and interpreted independent of all the entities supporting the project.

Supplemental Figures 1–3, Supplemental Tables 1–6, and Supplemental Methods are available from the “Supplementary data” link in the online posting of the article and from the same link in the online table of contents at https://academic.oup.com/ajcn/.

Abbreviations used: ANOSIM, analysis of similarities; dbRDA, distance-based redundancy analysis; FPQ, food propensity questionnaire; HFC, healthy food choices; KO, Kyoto Encyclopedia of Genes and Genomes orthology; MaAsLin, multivariate association with linear models; PCA, principal coordinate analysis; PERMANOVA, permutational multivariate analysis of variance.
