# Supplementary material for: Associations of healthy food choices with gut microbiota profiles
Source: Am J Clin Nutr. 2021 May 21;114(2):605–16. doi: 10.1093/ajcn/nqab077 (PMC8326043; doi:10.1093/ajcn/nqab077)
Supplement: nqab077_Supplemental_Files [file nqab077_supplemental_files.zip › supplementary_methods_1.pdf]

Supplementary Methods 1; On-line Supplementary Material; Associations of healthy food choices with gut microbiota profiles; Koponen et al.

Listings of ATC codes of medicines that were filtered out or adjusted for.

Systemic antimicrobial medicines

J	ANTIINFECTIVES FOR SYSTEMIC USE
J01	ANTIBACTERIALS FOR SYSTEMIC USE
J01A	TETRACYCLINES
J01B	AMPHENICOLS
J01C	BETA-LACTAM ANTIBACTERIALS, PENICILLINS
J01D	OTHER BETA-LACTAM ANTIBACTERIALS
J01E	SULFONAMIDES AND TRIMETHOPRIM
J01F	MACROLIDES, LINCOSAMIDES AND STREPTOGRAMINS
J01G	AMINOGLYCOSIDE ANTIBACTERIALS
J01M	QUINOLONE ANTIBACTERIALS
J01R	COMBINATIONS OF ANTIBACTERIALS
J01X	OTHER ANTIBACTERIALS
J02	ANTIMYCOTICS FOR SYSTEMIC USE
J04	ANTIMYCOBACTERIALS
J05	ANTIVIRALS FOR SYSTEMIC USE
J06	IMMUNE SERA AND IMMUNOGLOBULINS

Medicines with potentially microbiome altering effects

A	ALIMENTARY TRACT AND METABOLISM
A06	DRUGS FOR CONSTIPATION
A07	ANTIDIARRHEALS, INTESTINAL ANTIINFLAMMATORY/ANTIINFECTIVE AGENTS
A08	ANTIOBESITY PREPARATIONS, EXCL. DIET PRODUCTS
A10	DRUGS USED IN DIABETES
A10A	INSULINS AND ANALOGUES
A10B	BLOOD GLUCOSE LOWERING DRUGS, EXCL. INSULINS
A10BA	Biguanides
A10BA01	phenformin
A10BA02	metformin
A10BA03	buformin
A10BB	Sulfonylureas
A10BC	Sulfonamides (heterocyclic)
A10BD	Combinations of oral blood glucose lowering drugs
A10BD01	phenformin and sulfonylureas
A10BD02	metformin and sulfonylureas
A10BD03	metformin and rosiglitazone
A10BD04	glimepiride and rosiglitazone
A10BD05	metformin and pioglitazone
A10BD06	glimepiride and pioglitazone
A10BD07	metformin and sitagliptin
A10BD08	metformin and vildagliptin
A10BD09	pioglitazone and alogliptin
A10BD10	metformin and saxagliptin
A10BD11	metformin and linagliptin
A10BD12	pioglitazone and sitagliptin
A10BD13	metformin and alogliptin
A10BD14	metformin and repaglinide
A10BD15	metformin and dapagliflozin
A10BD16	metformin and canagliflozin
A10BD17	metformin and acarbose
A10BD18	metformin and gemigliptin
A10BD19	linagliptin and empagliflozin
A10BD20	metformin and empagliflozin
A10BD21	saxagliptin and dapagliflozin
A10BD22	metformin and evogliptin
A10BF	Alpha glucosidase inhibitors
A10BG	Thiazolidinediones
A10BH	Dipeptidyl peptidase 4 (DPP-4) inhibitors
A10BJ	Glucagon-like peptide-1 (GLP-1) analogues
A10BK	Sodium-glucose co-transporter 2 (SGLT2) inhibitors
A10BX	Other blood glucose lowering drugs, excl. insulins
A10X	OTHER DRUGS USED IN DIABETES
B	BLOOD AND BLOOD FORMING ORGANS
C	CARDIOVASCULAR SYSTEM
C01	CARDIAC THERAPY
C02	ANTIHYPERTENSIVES
C10	LIPID MODIFYING AGENTS
C10A	LIPID MODIFYING AGENTS, PLAIN
C10AB	Fibrates
C10AC	Bile acid sequestrants
C10AD	Nicotinic acid and derivatives
C10AX	Other lipid modifying agents
C10B	LIPID MODIFYING AGENTS, COMBINATIONS
C10BA	HMG CoA reductase inhibitors in combination with other lipid modifying agents
C10BX	HMG CoA reductase inhibitors, other combinations
G	GENITO URINARY SYSTEM AND SEX HORMONES
G03	SEX HORMONES AND MODULATORS OF THE GENITAL SYSTEM
H	SYSTEMIC HORMONAL PREPARATIONS, EXCL. SEX HORMONES AND INSULINS
L	ANTINEOPLASTIC AND IMMUNOMODULATING AGENTS
N	NERVOUS SYSTEM
P	ANTIPARASITIC PRODUCTS, INSECTICIDES AND REPELLENTS
